# Supplementary material for: Learning curve and surgical outcome of robotic assisted colorectal surgery with ERAS program
Source: Sci Rep. 2022 Nov 29;12:20566. doi: 10.1038/s41598-022-24665-w (PMC9709162; doi:10.1038/s41598-022-24665-w)
Supplement: Supplementary file 1 — Supplementary Information 1. [file 41598_2022_24665_MOESM1_ESM.zip › LSC Raw data-Ñ[▒K/067/O_ñw╝╨░O▒Kñσ.pdf]

正式摘要(電子簽章：000000186016267)

請選擇...reporter

臺中榮民總醫院

Operation Note

[redacted]

- 手術日期：2020/02/14
- 手術醫師：VS 蔣鋒帆，CR 黃佢霖，R 楊涵中，R 邱俊凱
- Anesthesia : GE
- Position : Supine
- Aseptic agents : Betadine Alcohol

Diagnosis

- Pre-operative diagnosis : Malignant tumor of cecum
- Post-operative diagnosis : Ditto.

Surgical Indication

- For oncological resection

Operation

- Laparoscopic right colectomy and anastomosis

Finding

- Operative findings:
  1. A 3x4cm Borrmann type III ulcerative tumor over cecum, which invaded through muscular layer, but didn't penetrate through serosa. No enlarged lymph nodes at mesocolon.
  2. No other gross tumor or polyp was found.
  3. There was no visible liver metastasis or visible peritoneal seeding.

  Operative methods:
  1. Laparoscopic right hemicolectomy with side-to-side anastomosis, GIA 75/4.5*2, re-inforcement with silk (+).
  2. Vessel ligation : ileo-colic, right colic, and right branch of middle colic vessels.
  3. Cut margin: proximal (ileum) 10 cm, (colon) 5 cm, distal 20 cm.
  4. Repair of mesentery defect: done.
  5. A 12mm Penrose drainage placed at Morrison's pouch.

Procedure

- General endotracheal anesthesia was induced and then the patient was placed in the supine position. Preoperative antibiotics were given. A Foley catheter and nasogastric tube were placed. The abdomen was prepped and draped in standard sterile fashion. A small vertical incision was made below the umbilicus and a 12mm trocar was inserted into the peritoneal cavity by open method. Pneumoperitoneum was established with CO2 inflation. Laparoscope was inserted and then a 12mm trocar were inserted over LUQ of abdomen. Two 5mm trocars were inserted over RUQ and LLQ of abdomen. The liver, omentum and peritoneum were inspected for evidence of metastatic disease. No metastatic disease was noted.

  The small bowel was inspected and retracted to downward by grasper. The peritoneum overlying the mesentery was then scored with electrocautery and the ileocolic artery was identified, ligated with endoclips, and transected. The remaining vessels to the cecum were also divided and ligated. Using electrocautery, omentum was freed from the transverse colon and lesser sac was entered. The colon was freed from its peritoneal attachments along the line of Toldt from the cecum to the hepatic flexure. Hemostasis was checked in the operative field. Cecum, ascending colon and hepatic flexure

- were mobilized and then laparoscope was removed. One midline incision was made at peri-umbilical region. The ascending colon was delivered into the incision and points of transection were selected proximally and distally. Side to side anastomosis was performed with endo GIA 75/3.85. Interrupted 3-0 silk Lambert sutures were used to re-inforcement at outer layer. The anastomosis was checked and found to be intact and widely patent. One 12mm penrose draian was placed at sub-hepatic region. The fascia was closed with interrupted sutures of No.1 Vicryl. The fascia was closed with No.1 Vickyl and the skin was closed with 3-0 Nylon. The patient tolerated the procedure well and was brought to the POR in stable condition
- Estimated blood loss: 350 c.c
- 器械數量: 正確
- 紗布數量: 正確

Image

- PAINT
  - colon & rectum

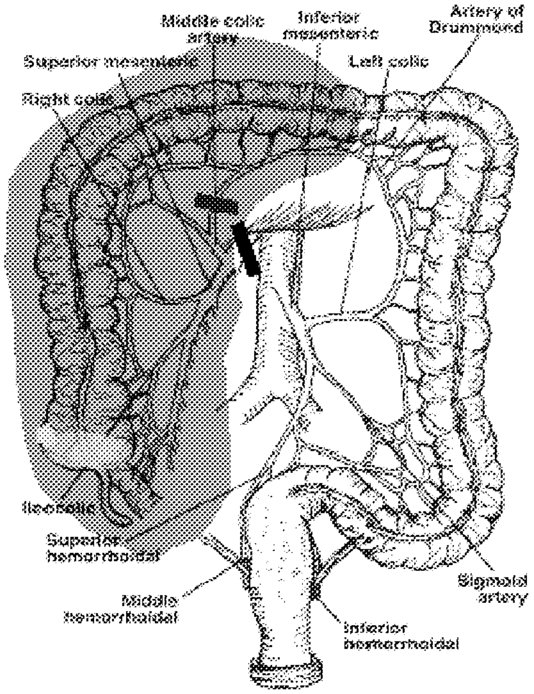

- 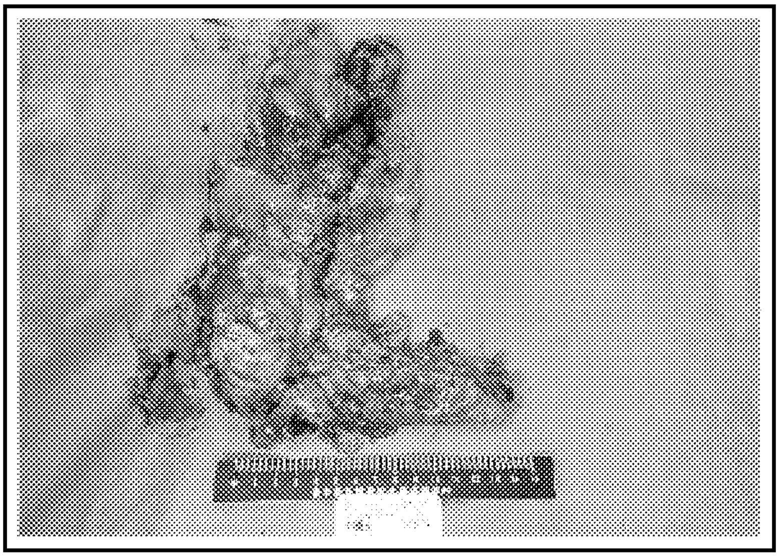

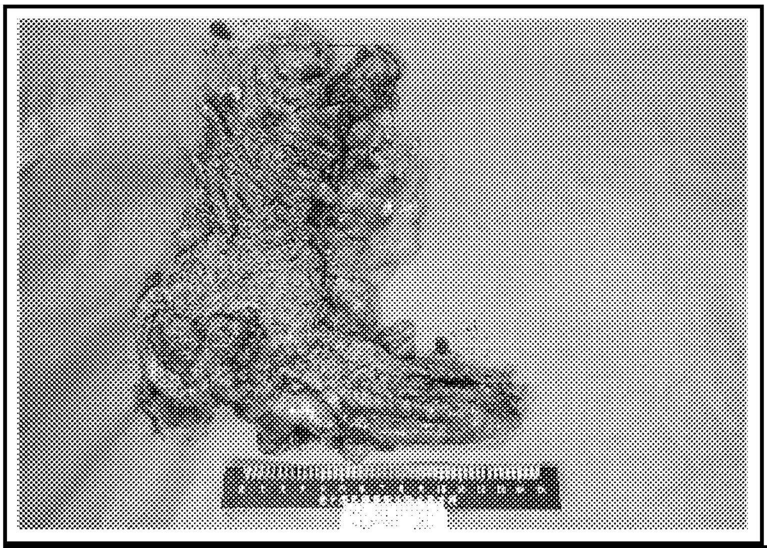

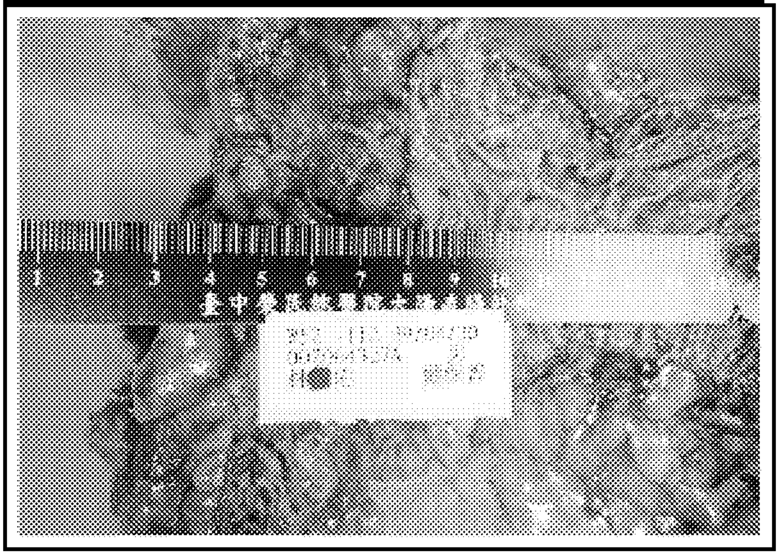

編輯主任 2062F蔣鋒帆、主治 2286H黃俋霖

正式摘要(電子簽章：000000186016267)

請選擇...reporter

doctorRank:B
